# Sudden death of COVID-19 patients in Wuhan, China: A retrospective cohort study

**DOI:** 10.7189/jogh.11.05006

**Published:** 2021-03-27

**Authors:** Nan Yang, Kunming Tian, Meng Jin, Xu Zhang, Fengqin Zhang, Xiuquan Shi, Xiaoyang Wang, Siyuan Niu, Jing Shi, Ke Hu, Kui Liu, Ping Peng, Ying Wang, Huilan Zhang, Jianbo Tian

**Affiliations:** 1 Department of Epidemiology and Biostatistics, Key Laboratory for Environment and Health, School of Public Health, Tongji Medical College, Huazhong University of Sciences and Technology, Wuhan, China; 2 Department of Respiratory and Critical Care Medicine Tongji Hospital, Tongji Medical College, Huazhong University of Science and Technology, Wuhan, China; 3 Institute of Reproductive Health, Tongji Medical College, Huazhong University of Science and Technology, Wuhan, China; 4 Department of Respiratory and Critical Care Medicine, Renmin Hospital of Wuhan University, Wuhan, China; 5 Key Laboratory of Environment and Health, Ministry of Education & Ministry of Environmental Protection, and State Key Laboratory of Environmental Health (Incubating), School of Public Health, Tongji Medical College, Huazhong University of Science and Technology, Wuhan, China; 6 Department of Virology, Wuhan Centers for Disease Prevention and Control, Wuhan, China; 7 Department of Oncology, Tongji Hospital, Tongji Medical College, Huazhong University of Science and Technology, Wuhan, China; 8 Department of Epidemiological and health statistics, School of Public Health, Zunyi Medical University, Zunyi, China

## Abstract

**Background:**

In December 2019, coronavirus disease 2019 (COVID-19) emerged in Wuhan city and rapidly spread throughout China. So far, it has caused ~ 4000 deaths in this country. We aimed to systematically characterize clinical features and determine risk factors of sudden death for COVID-19 patients.

**Methods:**

Deceased patients with COVID-19 in Tongji hospital from January 22 to March 23, 2020 were extracted. Patients who died within 24 hours after admission were identified as sudden deaths, and the others formed non-sudden deaths. The differences in clinical characteristics between the two groups were estimated. Risk factors associated with sudden deaths were explored by logistic regression.

**Results:**

281 deceased patients were enrolled in this study. Sudden death occurred in 28 (10.0%) patients, including 4 (14.3%) admitted to the intensive care unit. Fatigue was more common in sudden deaths (11, 47.8%) than in non-sudden deaths (40, 17.2%). Both the count and percentage of eosinophils were lower in sudden deaths than that in non-sudden deaths (*P* = 0.006 and *P* = 0.004). Compared with non-sudden deaths, sudden deaths had higher plasma levels of procalcitonin, C-reactive protein, D-dimer, alanine aminotransferase, aspartate aminotransferase, gamma-glutamyl transferase, lactate dehydrogenase, alkaline phosphatase and N-terminal pro-brain natriuretic peptide. There were not significant differences in gender, age, chest CT image features and comorbidities observed.

**Conclusions:**

The differences between the two groups suggested more severe systemic inflammation, multi-organ dysfunction, especially impaired liver and heart function in COVID-19 patients who died suddenly after admission. More researches are needed to verify these points.

In December 2019, a series of pneumonia cases of unknown cause were identified in Wuhan, China [1]. This pneumonia, later named as coronavirus disease 2019 (COVID-19) by WHO, was caused by a novel enveloped RNA betacoronavirus, SARS-CoV-2 [2]. Owing to the high infectivity and pathogenicity of the virus, COVID-19 has rapidly spread worldwide [3,4].

Case fatality rate was assessed as high as 1%, which is much greater than seasonal influenza at about 0.1% [5]. In China, the case fatality rate was 17.3% in the early stages of the outbreak and reduced over time to 0.7% after 1 February [6]. With the improvement of monitoring measures and medical treatment methods, the survival rate of patients has been greatly improved. However, there were still a small number of patients died within a short period of time after admission, in other words, sudden death. As no vaccine or specific antiviral therapy against COVID-19 has been proven to be effective, it is beneficial to implement supportive therapy to relieve symptoms and protect multiple organ functions. Treating patients at risk of sudden death promptly is essential to reduce mortality.

In this study, we collected data of deceased patients with COVID-19 in Tongji Hospital (Wuhan, China). By comprehensively evaluating the demographic, clinical, radiological, and laboratory characteristics between patients who died within 24 hours after admission and those who died over 24 hours after admission, we hope to find valuable markers related to sudden death.

## METHODS

### Patients

This single-center, retrospective study was performed at Tongji Hospital (Wuhan, China), which is a designated hospital to treat patients with COVID-19. All patients in this study were diagnosed with COVID-19 on the basis of World Health Organization interim guidance [7], and died between January 22 and March 23, 2020. patients who died within 24 hours after admission were defined as sudden deaths, while those who died more than 24 hours after admission were defined as non-sudden deaths. In total, 281 deceased patients with COVID-19 were included, consisting of 28 sudden deaths and 253 non-sudden deaths. This study was approved by the Ethics Committee of Tongji Hospital, Tongji Medical College, Huazhong University of Science and Technology.

### Laboratory procedures

Methods for laboratory confirmation of SARS-CoV-2 infection are standardized. Pharyngeal swabs were collected from patients and placed into tubes with virus preservation solution. Total RNA was extracted within two hours using respiratory sample RNA isolation kits (Pfizer or BioGerm, Shanghai, China). Two target genes, open reading frame 1ab (ORF1ab) and nucleocapsid protein (N), were simultaneously amplified and tested by real time RT-PCR assay, which was conducted using SARS-Cov-2 nucleic acid detection kits (BioGerm, Shanghai, China) according to the manufacturer’s protocol. The reaction mixture contained 12 μL of reaction buffer, 4 μL of enzyme solution, 4 μL of ORF1ab/N primers solution, 3 μL of RNase-Free Water, and 2 μL of RNA template. The RT-PCR reaction was under the following conditions: incubation at 50°C for 15 minutes and 95°C for 5 minutes, and 40 cycles of denaturation at 95°C for 10 seconds and fluorescence signal acquisition at 55°C for 45 seconds. Results were defined as negative (cycle threshold value >38 or not detected), positive (amplification curve was s-shaped, and cycle threshold value ≤35) and suspicious (amplification curve was s-shaped, and 35<cycle threshold value ≤38). In addition, nucleic acid test positive interpretation criteria are divided into two aspects: first, in the same specimen, ORF1ab and N genes were tested positive at the same time; second, the ORF1ab or N gene was positive in two different samples of the same patient. These criteria are based on the recommendation by the National Institute for Viral Disease Control and Prevention (China).

Other laboratory tests included complete blood count, serum biochemical tests (including liver and kidney function, creatine kinase, lactate dehydrogenase, and electrolytes), coagulation profile and cytokine tests. Chest CT scan were also done for inpatients. Frequency of examinations was determined by the physicians.

### Data collection

Demographic, clinical, radiological and laboratory characteristics and treatment data were collected from electronic medical records. If a patient received a same examination more than once, we only extracted the first result of the examination. All data were reviewed independently by two researchers.

### Statistical analysis

Categorical variables were presented as numbers and percentages. Continuous variables were presented as mean and standard deviation (SD) if they were normally distributed, or median and interquartile range (IQR) if they were not. Means for continuous variables were compared using independent sample *t* tests. Medians for continuous variables were compared using Wilcoxon rank sum tests. Categorical variables were compared using χ^2^ or Fisher exact tests. Univariable and multivariable logistic regression models were employed to explore risk factors associated with sudden death of COVID-19 patients. In multivariable logistic regression models, factors including age, sex and each comorbidity were adjusted. All tests were two sided. *P* values less than 0.05 were defined as statistically significant. IBM SPSS Statistics 22 (IBM Corp, Armonk NY, USA) was employed for all analyses.

## RESULTS

### Demographic features, clinical comorbidities and symptoms

The median age of all patients was 69.0 (IQR = 62.0-77.0). The ratio of male to female was 2.1. Most patients had comorbidities, of which hypertension was the most common type (38.8%), followed by diabetes (14.2%) and coronary heart disease (11.4%). There were not significant differences in age, gender and clinical comorbidities between sudden and non-sudden deaths. Fever was the most prevalent symptom in both sudden deaths (18, 78.3%) and non-sudden deaths (177, 76.0%), but the proportions of patients in the two groups were not comparable. Besides, for analyses on symptoms including dyspnea, diarrhea, chest tightness, chills, poor appetite, muscle pain, headache and vertigo, no significant differences were detected. Cough and expectoration were more common in non-sudden deaths (154, 66.1% and 102, 43.8%) than in sudden deaths (7, 30.4%, and 4, 17.4%). Fatigue was much more common in sudden deaths (11, 47.8%) than in non-sudden deaths (40, 17.2%). Results are shown in **Table 1**.

**Table 1:** Clinical characteristics, radiographic findings and treatments of the study patients*

Indicators	Total	Non-sudden deaths	Sudden deaths	*P* value
**Demographic features:**	**N = 281**	**N = 253**	**N = 28**	
Age, years	69.0 (62.0-77.0)	69.0 (62.0-78.0)	68.0 (62.0-74.5)	0.66
Sex:				
-Male	191 (68.0%)	172 (68.0%)	19 (67.9%)	1
-Female	90 (32.0%)	81 (32.0%)	9 (32.1%)	
**Comorbidities:†**	**N = 281**	**N = 253**	**N = 28**	
Hypertension	109 (38.8%)	99 (39.1%)	10 (35.7%)	0.883
Diabetes	40 (14.2%)	35 (13.8%)	5 (17.9%)	0.569
CHD	32 (11.4%)	29 (11.5%)	3 (10.7%)	1.000
Cancer	23 (8.2%)	18 (7.1%)	5 (17.9%)	0.064
Cerebral infarction	12 (4.3%)	11 (4.3%)	1 (3.6%)	1.000
Pulmonary tuberculosis	9 (3.2%)	8 (3.2%)	1 (3.6%)	1.000
Chronic bronchitis	8 (2.8%)	8 (3.2%)	0 (0.0%)	1.000
COPD	4 (1.4%)	4 (1.6%)	0 (0.0%)	1.000
Hepatitis and liver cirrhosis	7 (2.5%)	6 (2.4%)	1 (3.6%)	0.524
Others	75 (26.7%)	66 (26.1%)	9 (32.1%)	0.644
**Initial symptoms:†**	**N = 256**	**N = 233**	**N = 23**	
Fever	195 (76.2%)	177 (76.0%)	18 (78.3%)	1
Dyspnea	129 (50.4%)	118 (50.6%)	11 (47.8%)	0.969
Cough	161 (62.9%)	154 (66.1%)	7 (30.4%)	0.002
Expectoration	106 (41.4%)	102 (43.8%)	4 (17.4%)	0.026
Diarrhea	59 (23.0%)	53 (22.7%)	6 (26.1%)	0.918
Fatigue	51 (19.9%)	40 (17.2%)	11 (47.8%)	0.001
Chest tightness	41 (16.0%)	35 (15.0%)	6 (26.1%)	0.227
Chills	25 (9.8%)	23 (9.9%)	2 (8.7%)	1.000
Poor appetite	21 (8.2%)	19 (8.2%)	2 (8.7%)	1.000
Muscle pain	15 (5.9%)	12 (5.2%)	3 (13.0%)	0.141
Headache	13 (5.1%)	12 (5.2%)	1 (4.3%)	1.000
Vertigo	19 (7.4%)	18 (7.7%)	1 (4.3%)	1.000
Others	20 (7.8%)	18 (7.7%)	2 (8.7%)	0.697
**CT findings:**	**N = 64**	**N = 60**	**N = 4**	
Ground-glass opacity	37 (57.8%)	36 (60.0%)	1 (25.0%)	0.302
Patchy shadows	48 (75.0%)	44 (73.3%)	4 (100.0%)	0.564
Fibrous stripes	9 (14.1%)	9 (15.0%)	0 (0.0%)	1.000
Pleural thickening	27 (42.2%)	25 (41.7%)	2 (50.0%)	1.000
Nodules	6 (9.4%)	5 (8.3%)	1 (25.0%)	0.332
Lymphadenia	28 (43.8%)	27 (45.0%)	1 (25.0%)	0.625
Bilateral pulmonary	64 (100.0%)	60 (100.0%)	4 (100.0%)	1.000
Right lung	6 (9.4%)	5 (8.3%)	1 (25.0%)	0.332
Left lung	6 (9.4%)	5 (8.3%)	1 (25.0%)	0.332
**Treatment:**	**N = 281**	**N = 253**	**N = 28**	
Antiviral therapy	126 (44.8%)	113 (44.7%)	13 (46.4%)	1
Antibiotic therapy	272 (96.8%)	246 (97.2%)	26 (92.9%)	0.223
Use of immune globulin	163 (58.0%)	155 (61.3%)	8 (28.6%)	0.002
Use of corticosteroid	252 (89.7%)	230 (90.9%)	22 (78.6%)	0.053
Renal-replacement therapy	102 (36.3%)	82 (32.4%)	20 (71.4%)	<0.001
Oxygen support:				
-Nasal cannula	100 (35.6%)	82 (32.4%)	18 (64.3%)	0.002
-Non-invasive ventilation	74 (26.3%)	68 (26.9%)	6 (21.4%)	0.693
-Invasive ventilation	105 (37.4%)	103 (40.7%)	2 (7.1%)	0.001
ECOM	6 (2.1%)	6 (2.4%)	0 (0.0%)	1.000
Admission to ICU	147 (52.3%)	143 (56.5%)	4 (14.3%)	<0.001

CHD – coronary heart disease, COPD – chronic obstructive pulmonary disease, ECMO – extracorporeal membrane oxygenation, ICU – intensive care unit

*Continuous variables were described as median (IQR). *P* values were calculated by Wilcoxon rank sum test. Categorical variables were expressed as number (%). *P* values were calculated by Pearson χ^2^ test or Fisher exact test.

†Other comorbidities: cerebrovascular disease, chronic kidney disease, emphysema, hyperuricemia, atrial fibrillation, Parkinson disease, Alzheimer disease, anxiety, hyperthyroidism, hypothyroidism, systemic lupus erythematosus, peptic ulcer; Other initial symptoms: hemoptysis, stomachache, palpitation, anorexia, aversion.

### Radiographic features of chest CT scan findings

Chest CT images were collected from 64 patients. Ground-glass opacity, patchy shadows and pleural thickening were the most common characteristics in both sudden deaths (1, 25.0%; 4, 100.0%; and 2, 50.0%) and non-sudden deaths (36, 60.0%; 44, 73.3%; and 25, 41.7%). All patients had bilateral pulmonary injury. No significant differences were detected between the two groups. Results are shown in **Table 1**.

### Treatments

A majority of the patients (272, 96.8%) received antibiotic therapy, 44.8% received antiviral therapy, and 89.7% used corticosteroid. Compared with patients with sudden death, non-sudden deaths had a higher percentage of immune globulin use (61.3% vs 28.6%, *P* = 0.002), while lower percentage of renal-replacement therapy (32.4% vs 71.4%). Invasive mechanical ventilation was initiated in more non-sudden deaths than sudden deaths (40.7% vs 7.1%, *P* = 0.001). Extracorporeal membrane oxygenation was performed in 6 patients (2.1%). 147 patients were admitted to the intensive care unit (ICU), with a higher percentage among non-sudden deaths than sudden deaths (56.5% vs 14.3%). Results are shown in **Table 1**.

### Characteristics of laboratory examinations

For blood routine results, the levels of eosinophils count and the percentage of eosinophils were much lower in sudden deaths than in non-sudden deaths (0.002 ± 0.006 vs 0.025 ± 0.090 × 10^9^/L and 0.014 ± 0.052 vs 0.238 ± 0.719%). The percentage of monocytes was also lower in sudden deaths than in non-sudden deaths (3.30, IQR = 2.58-4.25 vs 4.90, IQR = 2.80-8.00 %), but the values were roughly within the normal physiological range. Neutrophils count and the percentage of neutrophils were much higher in sudden deaths than in non-sudden deaths (10.34, IQR = 7.70-15.83 vs 7.30, IQR = 4.49-11.54 × 10^9^/L and 89.15, IQR = 86.88-92.40 vs 86.70, IQR = 79.10-92.20 %). In terms of immune cells, the levels of lymphocytes, CD3^+^ T cells, CD3^+^CD4^+^ T cells, CD3^+^CD8^+^ T cells, CD3^-^CD16^+^CD56^+^ NK cells in both groups were lower than normal, but the differences between the two groups were not statistically significant.

Inflammatory examination results showed that the concentrations of procalcitonin and C-reactive protein (CRP) were much higher in sudden deaths than in non-sudden deaths (0.54, IQR = 0.36-1.11 vs 0.23, IQR = 0.12-0.88 ng/mL; and 151.35, IQR = 122.40-202.02 vs 100.65, IQR = 57.12-161.07 mg/L).

In coagulation examinations, sudden deaths showed longer prothrombin time, higher prothrombin time international normalized ratio and lower prothrombin activity when compared with non-sudden deaths (16.30, IQR = 14.90-20.18 vs 15.30, IQR = 14.10-16.70 seconds; 1.29, IQR = 1.16-1.72 vs 1.20, IQR = 1.09-1.35; and 67.50, IQR = 47.00-79.00 vs 76.00, IQR = 64.00-87.00 %). Besides, the levels of D-dimer were much higher in sudden deaths than that in non-sudden deaths (7.96, IQR = 2.93-21.00 vs 3.36, IQR = 1.31-14.16 ug/mL).

Compared with non-sudden deaths, sudden deaths had higher levels of alanine aminotransferase (39.50, IQR = 28.50-60.00 vs 24.00, IQR = 17.00-41.00 U/L), aspartate aminotransferase (60.00, IQR = 37.75-85.25 vs 40.00, IQR = 28.00-58.00 U/L), gamma-glutamyl transferase (50.50, IQR = 34.75-97.00 vs 38.00, IQR = 24.00-65.00 U/L), lactate dehydrogenase (619.00, IQR = 549.25-884.50 vs 489.00, IQR = 357.00-648.00 U/L), alkaline phosphatase (93.50, IQR = 69.75-166.00 vs 76.00, IQR = 59.00-102.00 U/L) and N-terminal pro-brain natriuretic peptide (2044.00, IQR = 980.50-6315.00 vs 843.00, IQR = 330.00-2401.00 pg/mL). There were no significant differences in other factors of laboratory examinations. Results are shown in **Table 2**.

**Table 2:** Laboratory findings of the study patients*

Indicators	Normal range	Total		Non-sudden deaths		Sudden deaths		*P* value
**inflammatory factors:**								
Procalcitonin, ng/mL	<0.05	N = 261	0.29 (0.12-0.89)	N = 240	0.23 (0.12-0.88)	N = 21	0.54 (0.36-1.11)	0.029
CRP, mg/L	<1.0	N = 276	105.60 (59.33-164.43)	N = 250	100.65 (57.12-161.07)	N = 26	151.35 (122.40-202.02)	0.001
**Cytokines:**								
IL-6, pg/mL	0.0-7.0	N = 202	61.34 (29.27-151.45)	N = 193	59.69 (26.56-141.90)	N = 9	146.80 (63.03-158.90)	0.145
IL-10, pg/mL	0.0-9.1	N = 199	10.30 (6.35-18.70)	N = 191	10.40 (6.30-18.55)	N = 8	9.25 (6.70-23.70)	0.895
IL-8, pg/mL	0.0-62.0	N = 200	28.40 (16.35-61.82)	N = 192	28.35 (16.15-63.52)	N = 8	30.55 (20.33-38.88)	0.884
IL-1β, pg/mL	0.0-5.0	N = 200	5.00 (5.00-6.93)	N = 192	5.00 (5.00-7.03)	N = 8	5.00 (5.00-5.00)	0.248
IL-2R, U/mL	223.0-710.0	N = 198	1148.0 (740.3-1615.0)	N = 190	1126.0 (731.8-1599.3)	N = 8	1340.5 (1067-1782.25)	0.346
**Blood routine:**								
Leucocytes count, ×10^9^/L	3.50-9.50	N = 281	8.91 (6.00-13.03)	N = 253	8.46 (5.73-12.89)	N = 28	11.27 (8.68-17.90)	0.011
Erythrocytes count, ×10^12^L	4.30-5.80	N = 281	4.17 (3.60-4.63)	N = 253	4.17 (3.61-4.62)	N = 28	4.19 (3.52-4.70)	0.822
Monocytes, %	3.0-10.0	N = 281	4.60 (2.70-7.50)	N = 253	4.90 (2.80-8.00)	N = 28	3.30 (2.58-4.25)	0.003
Monocytes count, ×10^9^/L	0.10-0.60	N = 281	0.40 (0.26-0.62)	N = 253	0.40 (0.27-0.63)	N = 28	0.42 (0.24-0.54)	0.753
Neutrophils, %	40.0-75.0	N = 281	87.10 (79.90-92.20)	N = 253	86.70 (79.10-92.20)	N = 28	89.15 (86.88-92.40)	0.036
Neutrophils count, ×10^9^/L	1.80-6.30	N = 281	7.75 (4.53-11.63)	N = 253	7.30 (4.49-11.54)	N = 28	10.34 (7.70-15.83)	0.006
Eosinophils, %	0.4-8.0	N = 281	0.00 (0.00-0.10)	N = 253	0.00 (0.00-0.10)	N = 28	0.00 (0.00-0.00)	0.004
		N = 281	0.215 ± 0.686	N = 253	0.238 ± 0.719	N = 28	0.014 ± 0.052	
Eosinophils count, ×10^9^/L	0.02-0.52	N = 281	0.00 (0.00-0.01)	N = 253	0.00 (0.00-0.01)	N = 28	0.00 (0.00-0.00)	0.006
		N = 281	0.023 ± 0.086	N = 253	0.025 ± 0.090	N = 28	0.002 ± 0.006	
Basophils, %	0.0-1.0	N = 281	0.10 (0.00-0.20)	N = 253	0.10 (0.00-0.20)	N = 28	0.10 (0.08-0.12)	0.545
Basophils count, ×10^9^/L	0.00-0.10	N = 281	0.01 (0.00-0.02)	N = 253	0.01 (0.00-0.02)	N = 28	0.01 (0.01-0.02)	0.423
Hemoglobin, g/L	130.0-175.0	N = 281	128.00 (112.00-143.00)	N = 253	128.00 (111.00-143.00)	N = 28	122.00 (112.75-142.25)	0.761
Platelets count, ×10^9^/L	125.0-350.0	N = 281	159.00 (111.00-224.00)	N = 253	161.00 (112.00-224.00)	N = 28	147.00 (87.75-223.50)	0.580
**Lymphocytes:**								
Lymphocytes, %	20.0-50.0	N = 281	7.10 (4.10-12.20)	N = 253	7.20 (4.10-12.60)	N = 28	6.90 (4.33-9.12)	0.289
Lymphocytes count, ×10^9^/L	1.10-3.20	N = 281	0.63 (0.44-0.85)	N = 253	0.61 (0.43-0.84)	N = 28	0.66 (0.51-0.90)	0.23
CD3^+^ T cell count/μL	690-2540	N = 54	276.50 (132.75-408.50)	N = 49	269.00 (130.00-404.00)	N = 5	379.00 (332.00-448.00)	0.395
CD3^+^CD4^+^ T cell count/μL	410-1590	N = 54	170.50 (93.25-257.50)	N = 49	166.00 (93.00-243.00)	N = 5	264.00 (253.00-275.00)	0.257
CD3^+^CD8^+^ T cell count/μL	190-1140	N = 54	62.00 (29.25-127.00)	N = 49	61.00 (29.00-124.00)	N = 5	110.00 (56.00-158.00)	0.447
CD4^+^/CD8^+^ T cell count/μL	1.02-1.94	N = 54	2.86 (1.82-4.72)	N = 49	2.97 (1.83-4.79)	N = 5	2.40 (1.79-3.95)	0.633
CD3^-^CD16^+^CD56^+^ NK cell count/μL	88-64	N = 54	36.50 (16.00-74.75)	N = 49	35.00 (16.00-74.00)	N = 5	43.00 (26.00-114.00)	0.665
CD19^+^ B cell count/μL	77-736	N = 54	73.50 (40.25-143.00)	N = 49	69.00 (40.00-130.00)	N = 5	168.00 (75.00-229.00)	0.257
**Coagulation profiles:**								
APTT, s	26.0-42.0	N = 250	40.25 (36.23-46)	N = 229	40.10 (36.10-46.00)	N = 21	42.10 (38.00-48.30)	0.309
PT, s	10.0-15.0	N = 281	15.30 (14.30-16.90)	N = 253	15.30 (14.10-16.70)	N = 28	16.30 (14.90-20.18)	0.016
PT-INR	0.8-1.2	N = 281	1.20 (1.10-1.37)	N = 253	1.20 (1.09-1.35)	N = 28	1.29 (1.16-1.72)	0.017
D-dimer, ug/mL	<0.5	N = 275	4.10 (1.39-15.41)	N = 247	3.36 (1.31-14.16)	N = 28	7.96 (2.93-21.00)	0.004
Fibrinogen, g/L	2.0-4.0	N = 248	4.85 ± 2.08	N = 227	4.88 ± 2.08	N = 21	4.57 ± 2.18	0.510
PTA, %	70.0-140.0	N = 281	75.00 (62.00-86.00)	N = 253	76.00 (64.00-87.00)	N = 28	67.50 (47.00-79.00)	0.019
TT, s	11.0-17.0	N = 248	17.00 (15.80-19.10)	N = 228	17.10 (15.88-19.10)	N = 20	16.45 (15.75-18.38)	0.545
**Electrolytes:**								
Potassium, mmol/L	3.50-5.30	N = 281	4.33 (3.89-4.84)	N = 253	4.33 (3.87-4.84)	N = 28	4.29 (3.92-4.85)	0.89
Sodium, mmol/L	137.00-147.00	N = 281	138.80 (135.40-142.70)	N = 253	138.50 (134.80-142.40)	N = 28	141.25 (137.10-144.40)	0.054
Chloridion, mmol/L	90.00-110.00	N = 281	100.20 (96.20-104.30)	N = 253	100.00 (96.20-104.00)	N = 28	102.00 (97.17-106.10)	0.21
Calcium, mmol/L	2.11-2.52	N = 281	2.06 (1.99-2.14)	N = 253	2.06 (1.99-2.15)	N = 28	2.04 (1.97-2.09)	0.196
PHOS, mmol/L	0.85-1.51	N = 194	0.96 (0.75-1.28)	N = 189	0.96 (0.75-1.28)	N = 5	0.89 (0.77-1.43)	0.799
Magnesium, mmol/L	0.73-1.06	N = 195	0.91 (0.82-1.02)	N = 190	0.90 (0.82-1.01)	N = 5	0.93 (0.87-1.03)	0.490
**Organ damage index:**								
ALT, U/L	≤41	N = 281	27.00 (18.00-42.00)	N = 253	24.00 (17.00-41.00)	N = 28	39.50 (28.50-60.00)	0.001
AST, U/L	≤40	N = 281	41.00 (29.00-59.00)	N = 253	40.00 (28.00-58.00)	N = 28	60.00 (37.75-85.25)	0.003
GGT, U/L	6-42	N = 281	39.00 (25.00-69.00)	N = 253	38.00 (24.00-65.00)	N = 28	50.50 (34.75-97.00)	0.031
Total bilirubin, μmol/L	≤26	N = 281	12.30 (9.00-18.70)	N = 253	12.30 (9.00-18.70)	N = 28	12.00 (8.75-18.18)	0.917
Direct bilirubin, μmol/L	≤8.0	N = 280	6.15 (4.40-9.60)	N = 252	6.10 (4.40-9.30)	N = 28	6.30 (4.42-10.40)	0.630
Indirect bilirubin, μmol/L	≤16.8	N = 278	5.95 (4.10-8.40)	N = 250	5.95 (4.23-8.40)	N = 28	5.90 (3.53-8.25)	0.747
ALB, g/L	35-52	N = 280	31.10 (28.00-34.20)	N = 252	31.30 (27.98-34.32)	N = 28	29.75 (28.17-31.72)	0.157
GLO, g/L	20-35	N = 280	35.60 (31.48-39.05)	N = 252	35.65 (31.30-39.05)	N = 28	35.40 (33.40-39.10)	0.571
Total protein, g/L	64-83	N = 280	66.50 (61.82-70.82)	N = 252	66.45 (61.60-71.00)	N = 28	66.60 (62.50-68.80)	0.550
ALB/GLO	1.50-2.50	N = 280	0.86 (0.75-1.00)	N = 252	0.87 (0.76-1.01)	N = 28	0.84 (0.74-0.92)	0.305
Total bile acid, μmol/L	≤10	N = 177	3.40 (1.70-6.50)	N = 173	3.40 (1.70-6.50)	N = 4	3.35 (1.38-6.12)	0.726
Creatinine, μmol/L	45-84	N = 281	87.00 (67.00-114.00)	N = 253	86.00 (67.00-113.00)	N = 28	95.50 (64.75-119.00)	0.760
Urea, mmol/L	3.1-8.8	N = 279	8.30 (5.60-12.90)	N = 251	8.20 (5.60-12.80)	N = 28	9.74 (5.68-13.33)	0.405
Uric acid, μmol/L	142.8-339.2	N = 279	265.20 (194.00-371.50)	N = 251	265.20 (189.50-373.50)	N = 28	291.00 (222.75-335.25)	0.421
Total cholesterol, mmol/L	<5.18	N = 279	3.33 (2.83-3.96)	N = 251	3.33 (2.83-3.96)	N = 28	3.25 (2.82-3.69)	0.633
Triglyceride, mmol/L	0.56-1.7	N = 178	1.54 (1.18-2.19)	N = 171	1.53 (1.18-2.17)	N = 7	1.69 (1.12-2.19)	0.991
HDL-C, mmol/L	0.82-1.96	N = 177	0.78 ± 0.25	N = 170	0.78 ± 0.25	N = 7	0.76 ± 0.32	0.854
LDL-C, mmol/L	0.8-3.36	N = 176	1.90 (1.43-2.53)	N = 169	1.92 (1.43-2.54)	N = 7	1.86 (1.56-2.28)	0.797
Glucose, mmol/L	3.89-6.11	N = 275	7.84 (6.37-11.06)	N = 249	7.71 (6.32-10.95)	N = 26	8.95 (7.30-13.76)	0.067
LDH, U/L	135-225	N = 277	504.00 (364.00-669.00)	N = 251	489.00 (357.00-648.00)	N = 26	619.00 (549.25-884.50)	0.001
Alkaline phosphatase, U/L	35-105	N = 281	78.00 (60.00-107.00)	N = 253	76.00 (59.00-102.00)	N = 28	93.50 (69.75-166.00)	0.007
Choline esterase, U/L	4000-12000	N = 181	4947.48 ± 1618.62	N = 177	4948.79 ± 1616.65	N = 4	4889.50 ± 1965.47	0.942
NT-proBNP, pg/mL	<350	N = 252	888.50 (362.75-2566.50)	N = 233	843.00 (330.00-2401.00)	N = 19	2044.00 (980.50-6315.00)	0.008
Myoglobin, ng/mL	10-80	N = 178	237.20 (100.68-1200.00)	N = 170	237.20 (100.68-1200.00)	N = 8	258.10 (97.18-1150.88)	0.952
hs-cTnI, pg/mL	<0.1	N = 261	35.30 (11.20-194.70)	N = 239	31.10 (10.90-192.90)	N = 22	65.00 (24.17-246.02)	0.082
Creatine kinase, U/L	30-170	N = 192	129.00 (69.75-332.75)	N = 184	127.50 (68.75-328.75)	N = 8	290.00 (107.50-788.25)	0.153

CRP – C-reactive protein, IL-6 – interleukin 6, IL-10 – interleukin 10, IL-8 – interleukin 8, IL-1β - interleukin 1β, IL-2R – Interleukin 2 receptor, APTT – activated partial thromboplastin time, PT – prothrombin time, PT-INR – prothrombin time international normalized ratio, PTA – prothrombin activity, TT – thrombin time, ALT – glutamic-pyruvic transaminase, AST – glutamic-oxalacetic transaminase, GGT – gamma-glutamyl transferase, ABL – albumin, GLO – globulin, HDL-C – high density lipoprotein cholesterin, LDL-C – low density lipoprotein cholesterol, LDH – lactic dehydrogenase, NT-proBNP – N-terminal pro brain natriuretic peptide, hs-cTnI – high-sensitivity cardiac troponin I

*Continuous variables were described as median (interquartile range) or mean (± standard deviation). *P* values were calculated by Wilcoxon rank sum tests for skewed distributed data, and independent sample *t* tests for normal distributed data.

Furthermore, logistic models were employed to explore risk factors from laboratory examinations associated with the sudden death of COVID-19 patients. Both in univariate and multivariate logistic regression models, CRP (OR = 1.01, *P* = 0.003), percentage of monocytes (OR = 0.78, *P* = 0.004), percentage of neutrophils (OR = 1.07, *P* = 0.023), neutrophils count (OR = 1.09, *P* = 0.010), D-dimer (OR = 1.06, *P* = 0.011), prothrombin activity (OR = 0.98, *P* = 0.020), gamma-glutamyl transferase (OR = 1.00, 95% CI 1.00-1.01, *P* = 0.034) and alkaline phosphatase (OR = 1.01, *P* < 0.001) were identified as being associated with sudden death of COVID-19 patients. Results are shown in **Table 3**.

**Table 3:** Factors of laboratory examinations associated with the sudden death of COVID-19 patients

	Univariate logistic regression		Multivariate logistic regression *	
**Indicators**	**OR (95% CI)**	***P* value**	**OR (95% CI)**	***P* value**
**Inflammatory factors:**				
CRP, mg/L	1.01 (1.00-1.01)	**0.003**	1.01 (1.00-1.01)	**0.003**
**Blood routine:**				
Monocytes, %	0.78 (0.66-0.93)	**0.004**	0.76 (0.63-0.92)	**0.004**
Neutrophils, %	1.07 (1.01-1.13)	**0.023**	1.08 (1.02-1.15)	**0.015**
Neutrophils count, ×10^9^/L	1.09 (1.02-1.16)	**0.01**	1.10 (1.03-1.17)	**0.007**
**Coagulation profiles:**				
D-dimer, ug/mL	1.06 (1.01-1.11)	**0.011**	1.06 (1.01-1.11)	**0.014**
PTA, %	0.98 (0.96-1.00)	**0.02**	0.98 (0.96-1.00)	**0.032**
**Organ damage index:**				
GGT, U/L	1.00 (1.00-1.01)	**0.034**	1.00 (1.00-1.01)	**0.028**
Alkaline phosphatase, U/L	1.01 (1.01-1.02)	**<0.001**	1.01 (1.01-1.02)	**<0.001**

CRP – C-reactive protein, IgA – immunoglobulin A, FDP – fibrinogen degradation products, PTA – prothrombin activity, GGT – gamma-glutamyl transferase, LDH – lactic dehydrogenase, OR – odds ratio, CI – confidence interval

*Adjusted for age, sex, comorbidities including hypertension, diabetes, CHD, COPD, cerebral infarction, hepatitis and liver cirrhosis, pulmonary tuberculosis, chronic bronchitis, cancer and others.

## DISCUSSION

We report here a total of 281 deceased patients with COVID-19, and comprehensively described the major differences in clinical characteristics between sudden deaths and non-sudden deaths. The median age of deceased patients was 69.0 years. Compared with female, male sex was more predominant in deceased patients. Comorbidities were present in more than half of the deceased patients, with hypertension being the most common type, followed by diabetes and coronary heart disease. However, significant differences in age, sex and comorbidities between sudden deaths and non-sudden deaths were not observed, suggesting that although these factors are associated with death in patients with COVID-19 [5,8-10], they are not the cause of rapid deterioration and death in a short period of time. The incidence of symptoms including fever, dyspnea and diarrhea showed no significant differences between sudden deaths and non-sudden deaths. Fatigue was more common in sudden deaths, whereas dry cough and expectoration were more common in non-sudden deaths. Certain symptoms may be helpful for identifying the patients at risk of an acute death.

The vast majority of deceased patients showed bilateral pulmonary injury and patchy shadows was the most common finding on chest CT images on admission, but the radiographic findings did not differ between sudden deaths and non-sudden deaths. This indicates that chest CT scan is of little significance for predicting sudden death in critical period, and more meaningful laboratory indicators should be included. Deceased patients developed leukocytosis and lymphopenia, suggesting a possible secondary bacterial infection and cellular immune deficiency. It is worth noting that the decrease of eosinophils was more serious in sudden deaths, which is consistent with previous report that eosinophils may predict the outcome of COVID-19 progression [11]. Eosinophils could be an effective marker for monitoring the severity of COVID-19. We also reported that patients with high percentage of neutrophils or neutrophils count had an increased risk of sudden death, probably due to cytokine storm activated by neutrophils [8]. Higher serum levels of inflammatory marker, CRP, was identified as risk factors of sudden death with COVID-19 patients. This marker is important in immunity and immunopathology during virus infection [12-15]. Notably, significantly higher concentrations of alanine aminotransferase, aspartate aminotransferase, gamma-glutamyl transferase, lactate dehydrogenase, alkaline phosphatase and N-terminal pro-brain natriuretic peptide were observed in sudden deaths than in non-sudden deaths, indicating more impaired liver and heart function. In addition, compared with non-sudden deaths, sudden deaths had more severe changes in coagulation profiles, which also suggested more serious liver injury. These results indicated that the rapidly damaged liver and heart function was likely to cause sudden death of patients with COVID-19.

In this study, we could not prove that antiviral treatment was effective. Supportive therapy that protects important organs and eases the symptoms, like oxygen support, especially the comprehensive treatment and continuous care in the ICU, seemed be beneficial. We observed higher percentages of renal-replacement therapy and nasal cannula in sudden deaths than in non-sudden deaths, but we cannot conclude these treatments are futile to COVID-19 patients, as they were in severe condition when they received these treatments.

Since existing evidence is limited, our study of 281 deceased patients with COVID-19 represents the features of deaths and provides relevant clues to find the associations between sudden death of COVID-19 patients and potential risk factors. Additionally, the statistical analyses such as multivariable logistic regression models could adjust for confounders and minimize the bias. Also, several limitations should be considered. First, it was a retrospective, single-center study, not all radiographic or laboratory examinations were performed in all patients. Interpretation of our findings might be limited by the sample size. Larger cohort with more complete standardized data would make more sense. Second, patients were sometimes admitted to hospital late in illness. Data collected for each patient might be from different disease stages, which might lead to bias in clinical characteristics. Symptoms and comorbidities were self-reported by the patients as well as their family members, which might cause reporting bias. Third, patients with COVID-19 might have bacterial or other viral co-infection, which could affect the results of immune response. Forth, as it was a descriptive study, further mechanistic explanation still needs to be clarified. Despite that, our study demonstrated novel information about the characteristics of COVID-19 patients at risk of sudden death. This would help physicians to effectively identify patients with particularly poor prognosis on admission, and give necessary treatment in time, which may ultimately help to reduce the fatality rate.
